# Pre-conception counselling for key cardiovascular conditions in Africa: optimising pregnancy outcomes

**DOI:** 10.5830/CVJA-2016-017

**Published:** 2016

**Authors:** Liesl Zühlke, Letitia Acquah

**Affiliations:** Departments of Paediatric Cardiology and Medicine, Red Cross War Memorial Children’s and Groote Schuur Hospitals, Cape Town, South Africa; Department of Medicine, Division of Hospital Internal Medicine, Mayo Clinic Hospital, Saint Mary’s Campus, Rochester, Minnesota, USA

**Keywords:** pre-conceptual counselling OR counselling, Africa, sub-Saharan Africa OR Afric*

## Abstract

The World Health Organisation (WHO) supports pre-conception care (PCC) towards improving health and pregnancy outcomes. PPC entails a continuum of promotive, preventative and curative health and social interventions. PPC identifies current and potential medical problems of women of childbearing age towards strategising optimal pregnancy outcomes, whereas antenatal care constitutes the care provided during pregnancy. Optimised PPC and antenatal care would improve civil society and maternal, child and public health. Multiple factors bar most African women from receiving antenatal care. Additionally, PPC is rarely available as a standard of care in many African settings, despite the high maternal mortality rate throughout Africa. African women and healthcare facilitators must cooperate to strategise cost-effective and cost-efficient PPC. This should streamline their limited resources within their socio-cultural preferences, towards short- and long-term improvement of pregnancy outcomes.

This review discusses the relevance of and need for PPC in resource-challenged African settings, and emphasises preventative and curative health interventions for congenital and acquired heart disease. We also consider two additional conditions, HIV/AIDS and hypertension, as these are two of the most important co-morbidities encountered in Africa, with significant burden of disease. Finally we advocate strongly for PPC to be considered as a key intervention for reducing maternal mortality rates on the African continent.

## Abstract

The World Health Organisation (WHO) recently stated that four out of 10 women report that their pregnancies were unplanned. As a result, 40% of pregnancies miss the essential health interventions required prior to pregnancy. Despite the laudable gains achieved by some countries in the United Nations’ millennium development goal 5 target 5A, ‘Reduce by three-quarters, between 1990 and 2015, the maternal mortality ratio’, maternal morbidity remains a critical concern and public health issue in Africa.^1^ The WHO strongly supports the need for optimal pre-conception care (PCC) or counselling, followed by comprehensive antenatal care.^2^

PCC is defined as the continuum of promotive, preventative and curative health and social interventions.^3^ In addition to health interventions, other sectors and stakeholders need to be engaged to ensure universal access to PPC. PCC aims at improving the health status of prospective parents and reducing behaviours and individual and environmental factors that contribute to poor maternal and child health outcomes. Its ultimate aim is to improve maternal and child health, in both the short and long term.

It is important to note that although PCC aims primarily at improving maternal and child health, it brings health benefits to adolescents, women and men as individuals in their own right (not just as potential parents).^4^ Among others, PCC can improve a variety of important health outcomes including: reducing maternal and child mortality; preventing unintended pregnancies, perinatal complications, reducing the vertical transmission of HIV/STIs, and co-morbid infections such as rubella; and reducing the risk of type 2 diabetes mellitus and cardiovascular disease later in life. PPC identifies current and potential medical problems of women of childbearing age, in order to strategise optimal pregnancy outcomes.

The WHO has developed a package of PPC interventions that focuses on information and perspectives on important issues, target groups, delivery mechanisms and specific regional considerations. These are focused around 13 areas and provide an evidence-based package of interventions addressing the following areas: nutritional conditions, vaccine-preventable diseases, genetic conditions, environmental health, infertility/ subfertility, female genital mutilation, too early, unwanted and rapid successive pregnancies, sexually transmitted infections, HIV, interpersonal violence, mental health, psychoactive substance abuse, and tobacco use (Table 1).^2^

**Table 1:** Pre-conception care

*WHO package of evidenced-based interventions*	*Components of pre-conception care*	*Specific conditions addressed by pre-conception care only*
Nutritional conditions	Medical history	Conditions that need time to correct prior to conception
Genetic conditions	Psychosocial issues	Interventions not usually undertaken in pregnancy
Vaccine-preventable conditions	Physical examination	Intervention considered only because a pregnancy is planned.
Environmental health	Laboratory tests	Conditions that might change the choice/timing or method to conceive
Infertility/subfertility	Family history	Conditions requiring early post-conception pre-natal care
Female genital mutilation	Nutritional assessment	
Too early, unwanted and rapid successive pregnancies		
Sexually transmitted infections		
HIV		
Interpersonal violence		
Mental health		
Psychoactive substance use		
Tobacco use		

Adapted from: Preconception care to reduce maternal and childhood mortality and morbidity. Meeting report and packages of interventions: WHO HQ, February 2012; Preconception care: Greater New York Chapter of the March of Dimes Preconception Care Curriculum Working Group 2015.

It is clear that addressing non-medical and medical causes and correlates of maternal morbidity and mortality will optimise healthy pregnancy outcomes.^5^ Various authorities have studied key non-medical issues, namely, women’s education and family planning, which directly impact on the general welfare of childbearing women and enhance pregnancy outcomes.^6^

Of note is the importance of key collaborations and multisector engagement in order to devise a local strategy for PCC. Such a strategy would need to be informed by an assessment of the strengths and weaknesses of the PCC system in place. It will need to be supported by key stakeholders and partnerships to ensure political commitment, and it has to leverage on existing public health programmes. It would also need to be adapted to country priorities and target populations, while identifying service-delivery mechanisms within existing programmes. Innovative programmes have to be explored to highlight PCC. Consequently, adequate financial resources should be mobilised to support strategic implementation, monitoring and evaluation of viable PCC programmes.^2^

Having outlined the vision for PPC and the specific need within the African continent, we will focus our attention on some specific conditions requiring comprehensive PPC and assessment. Given the burden of disease of congenital, rheumatic and hypertensive heart disease, as well as HIV/AIDS, we will discuss these conditions by suggesting clear guidelines for clinicians caring for such patients, as well as strategies to improve outcomes relating to these conditions. Although we describe specific medical interventions to optimise health prior to pregnancy, the general evidence-based interventions should be the platform upon which these are based. These include screening for anaemia, nutritional supplementation (iron and folate), information, education and counselling, food supplementation, promoting exercise and a healthy diet, and family planning and child spacing (Table 2).

**Table 2:** Clinical pearls: planning pregnancy with certain medical conditions

*Medical condition*	*Preventative measures and supplementation*	*Contra-indications to pregnancy*	*Key points*
Congenital heart disease	Rubella vaccination	WHO IV risk score	Needs comprehensive risk assessment before pregnancy
Rheumatic heart disease	Primary prevention of group A streptococcus with penicillin	WHO IV risk score	Needs comprehensive risk assessment before pregnancy
	Institute secondary prevention with penicillin after a diagnosis of ARF/RHD		
Hypertension	Identify and treat secondary causes, treat sleep-disordered breathing, lifestyle changes	ACE inhibitors and ARBs	Normalise pre-pregnancy blood pressure
HIV	Treat co-morbidities	None	Avoid efavirenz if possible
			Optimise ART to maximal suppression of viral load
			Improved ART adherence
			Advise appropriate contraception
General	Screen for anaemia	As per examination	Information, education and counselling
	Food supplementation, iron and folate supplementation		Promote exercise and healthy diet
			Family planning and spacing
			Weight control
			Substance and tobacco control

## Systematic review

We performed a literature review of publications in PubMed, employing no language restriction, on the use of pre-conception counselling in Africa. Search terms included combinations of ‘((preconceptual[All Fields] AND (‘counselling’[All Fields] OR ‘counseling’[MeSH Terms] OR ‘counseling’[All Fields])))’ and ‘Africa OR sub-Saharan Africa’ or Afric*. We identified no previous studies that report pre-conception counselling in Africans.

This review responds to the need for pre-conception counselling in African women. It provides an overview of the need, details and goals of such counselling and then describes specific important conditions.

There are several studies detailing pre-conception counselling in different situations similar to the ones described. However, these are all from developed countries, therefore the findings cannot be generalised to the African context.

Our review highlights the need for multidisciplinary team approaches to pregnancy and for pre-conception clinics in specific key disease groups. We anticipate that this review will be an important resource for physicians, obstetricians and gynaecologists working in developing country settings.

## Congenital heart disease

The story of congenital heart disease is one of the major successes of medicine in the last 50 years. The vast majority of lesions are amenable to surgery and neonatal surgery is now the norm rather than the exception.^7^ Many women with congenital heart disease are currently in their childbearing years, and desire pregnancy to bear their own children; however, there is a startling difference in the situation in Africa.^8^

With very few specialised cardiothoracic centres in Africa, the majority of children requiring congenital heart surgery have no access to these centres.^9^ Adults with congenital heart disease in Africa fall into two categories, namely, those who are ‘postoperation’ or ‘post-intervention’, and adults with ‘previously undiagnosed’ congenital heart disease (recognised for the first time at pregnancy, or in early adulthood). The latter category is seldom encountered in the developed world. Both categories of women should be offered comprehensive PPC by a dedicated multidisciplinary team, because each category presents a unique set of cardiac and obstetric challenges, requiring an individualised assessment of risks and a carefully documented care plan.^10^

A large proportion of women attending cardio-obstetric clinics have documented congenital heart disease. A recent review of one clinic in Cape Town, South Africa, showed that almost a third (32%, 15 with previous operations) had congenital heart disease.^11^

Several scoring systems are used to risk stratify women contemplating pregnancy. The most commonly used are the cardiac disease in pregnancy (CARPREG) score, the ZAHARA (Zwangerschap bij Aangeboren Hartafwijking) score, and the WHO classification, which offer categories of risk.^12^ Class IV in the WHO score is extremely high risk, which contra-indicates pregnancy. Class IV includes: pulmonary hypertension, severe systemic ventricular dysfunction, dilated aortopathy and severe left-sided obstructive lesions.^6^ PPC must evaluate the potential risk posed by pregnancy to the woman, and include information regarding smoking, anticoagulation and anaemia, medication and recurrence of congenital heart defects in offspring.

Late presentation of left-to-right shunts in the African setting often results in Eisenmenger syndrome or pulmonary vascular disease, associated with cyanosis. Eisenmenger syndrome is associated with a high maternal and foetal risk, so the affected should be advised against pregnancy. Specialist counselling and contraceptive advice are essential to their care. Although treatment has improved, the maternal mortality rate remains in excess of 20% in developed countries, and probably closer to 50% in African settings.^13^

Unoperated tetralogy of Fallot is commonly found in Africa in association with cyanosis and severe right ventricular hypertrophy and significant antenatal risks. The most common left-sided lesion is coarctation of the aorta, which is usually repaired in the neonatal period. Because of late complications, such patients need lifelong surveillance due to high rates of hypertension, the need for re-intervention and decreased survival rates.^14^,^15^

Unoperated coarctation may cause severe hypertension, which can complicate pregnancy. Management of the hypertension may be difficult and reduction of maternal upper-body blood pressure may compromise the foeto-placental unit. These present significant challenges to the cardio-obstetric and cardioanaesthetic teams, so they are best managed before conception, with individualised patient-care plans, based on their anatomy and physiology.^16^

All patients with known cardiac disease should preferably be counselled before conception. Pre-pregnancy evaluation should include a comprehensive risk assessment for the mother and foetus, including medication use and information on heredity of the cardiac lesion. In cases of late diagnosis of congenital heart disease, combined with limited specialised cardiac resources, PPC is crucial to assessing pregnancy risks. Safe contraception options should be considered with a multidisciplinary management team. Continued attention should remain on the critical elements of PPC, such as nutritional support, family spacing and genetic conditions.

## Rheumatic heart disease

Rheumatic heart disease remains an endemic condition on the African continent, with an incidence of 27 per 100 000,^17^ and a prevalence of over 20 per 1 000 in sub-Saharan Africa.^18^,^19^ Moreover, recent studies demonstrate the severity of the disease in tertiary institutions in Africa, with the majority of cases presenting with established heart failure, atrial fibrillation and pulmonary hypertension.^20^,^21^

**Fig. 1.**
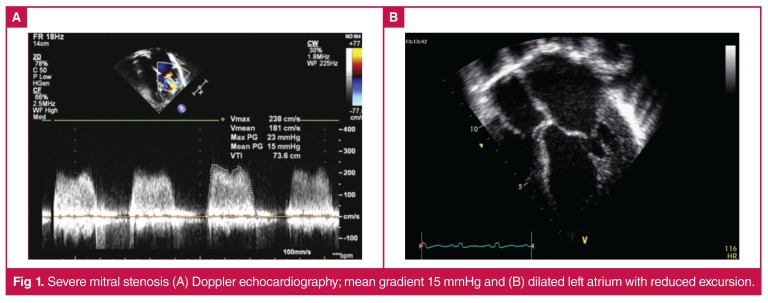
Severe mitral stenosis (A) Doppler echocardiography; mean gradient 15 mmHg and (B) dilated left atrium with reduced excursion.

The pathognomonic lesion in established rheumatic heart disease is mitral stenosis, which is associated with complications such as atrial fibrillation, stroke and death (Fig. 1). Valvular heart disease, especially stenotic valvular lesions, results in significant physiological effects during pregnancy, and is associated with maternal mortality and foetal loss.^22^,^23^ A previous study of 46 pregnant Senegalese women with rheumatic heart disease reported 17 maternal deaths (34%), six foetal deaths, and five therapeutic abortions.^24^

Severe mitral stenosis is classified as extremely high risk, therefore contra-indicating pregnancy. It is critical to evaluate all women of childbearing age with severe mitral stenosis, in order to provide family planning advice. In cases where pregnancy is strongly desired, pre-pregnancy interventions should be considered.^25^ Although mitral regurgitation is better tolerated during pregnancy, patients with severe symptomatic mitral regurgitation and impaired left ventricular function should be considered for timely surgery.^26^

Two final scenarios must be considered. The first scenario is the woman with a prosthetic heart valve desirous of pregnancy. Clear information on choices of anticoagulation therapy (e.g. heparin, warfarin or enoxaparin) during a potential pregnancy should be discussed with health professionals, with a clear plan to prevent complications and mortality.^27^ The second scenario is the patient with moderate mitral stenosis and a dilated left atrium, which increases the risk of stroke due to the lesion and the pregnant state.^28^ Once again, treatment options should be discussed prior to conception.

The evaluation of a woman with rheumatic heart disease prior to pregnancy should include taking a careful history and performing a detailed physical examination, 12-lead ECG and comprehensive echocardiogram, which should focus on the degree of left-sided valvular obstruction and systolic function. Finally, careful counselling to address both the general points of PCC and the specific risks of pregnancy (including the risk of miscarriage, early delivery, foetal losses and small for-gestationalage babies) should be paramount in this population.

## Hypertension

Blood pressure (BP) control before pregnancy should improve the effects of chronic hypertension on pregnancy outcomes. The weight of evidence indicates that chronically hypertensive women are at a higher risk of developing complications. Specific antihypertensive agents used by the chronically hypertensive woman should be titrated, discontinued or changed to other agents, in order to optimise her BP prior to pregnancy. Angiotensin converting enzyme inhibitors (ACEIs) and angiotensin receptor blockers (ARBs) are contra-indicated during pregnancy and must be discontinued when pregnancy is being planned.^29^-^32^

Whenever possible, pre-pregnancy BP should be normalised with lifestyle changes before pregnancy. These comprise: dietary changes (low-salt intake, increased intake of fresh fruits and vegetables), healthy weight modification to avoid obesity, and adherence to anti-hypertensive medications, which should improve health and pregnancy outcomes. When ACEIs or ARBs are discontinued before initiating a pregnancy, they could be replaced with other medications, e.g. hydralazine, alpha-methyldopa, nifedipine, diltiazem, labetalol or clonidine, if the benefits of the chosen drug outweigh its risks.

## HIV/AIDS

HIV/AIDS is a major public health concern and cause of death in many parts of Africa. The worst HIV/AIDS-affected people live in sub-Saharan Africa (SSA); 69% of all people living with HIV and 70% of all AIDS-related deaths in 2012 were from SSA,^33^ which had approximately 1.6 million new HIV infections and approximately 1.2 million AIDS-related deaths.

Globally, AIDS-related illnesses are the leading cause of death among childbearing women. SSA women are disproportionally affected; the percentage of those aged 15–24 years living with HIV is twice that of young men.^34^ HIV-infected women have many HIV-related medical and psychosocial issues, which may increase the risks of adverse HIV-pregnancy outcomes, perinatal and sexual transmission. While advances in HIV treatment and perinatal transmission have resulted in prolonged survival, improved quality of life and an increased number of pregnancies, PPC is required to optimise management to improve perinatal outcomes and minimise transmission risks (Table 2).

Key objectives for HIV/AIDS-related PPC are necessary. Firstly, maximal viral suppression should be achieved before conception. Detectable HIV plasma viral loads (PVL) and lack of effective antiretroviral treatment (ART) are associated with increased perinatal and sexual transmission.^35^ Furthermore, uncontrolled viral replication and non-adherence to ART cause viral resistance and overt disease. Sustaining high levels of adherence to ART with maximal viral suppression challenges resource-limited SSA, yet several programmes have demonstrated achievability.^36^

Secondly, PPC should explore the fertility desires of serodiscordant couples and offer options for safer conception. Early patient–provider communication about fertility goals could decrease peri-conception risks to HIV-uninfected partners.^37^ Although PPC is usually directed at women, exploring fertility goals with HIV-positive men in serodiscordant relationships could decrease peri-conceptional seroconversion in women.^35^

Exploring contraception needs informed, educated, reversible and irreversible contraception choices.^38^ An HIV-positive woman with excellent disease control and fertility control (reversible contraception) could have a healthy child at an optimal time, while preventing HIV transmission to her sexual partner and child.

Thirdly, PPC facilitates the appropriate choice of ART regimens. WHO guidelines recommend prescribing the same group of drugs to HIV-infected pregnant and non-pregnant women.^39^ Efavirenz has been associated with an increased risk of teratogenicity in recent studies conducted among infants exposed to efavirenz-containing regimens,^40^ however, WHO guidelines recommend the use of efavirenz as first-line therapy.^41^

Finally, PPC allows the assessment of common HIV-related co-morbidities before pregnancy, e.g. cardiovascular, kidney and liver diseases, cognitive dysfunction and mental health,^42^ malignancies and metabolic bone disease, and infections (viral hepatitis, HPV).^39^ A comprehensive assessment of metabolic and mental capacity before conception would improve general health-related outcomes (Table 1).

## Conclusion

Providing PPC in Africa is challenging at best. Due to the complexities barring access to PPC, the task of providing such care should be shared corporately among all healthcare providers who may have any appreciable encounter with women of childbearing age. There should be a concerted effort to position PCC as a public health intervention for maternal and child health, and it should aim at improving the general health status of women beyond perinatal care.

Public health educational campaigns should target at-risk groups to discuss the importance of reducing adverse pregnancy outcomes in order to optimise PPC. Beneficiaries and indirect stakeholders of the advantages of improved pregnancy outcomes should endeavour to provide cost-efficient and cost-effective PPC, within their resource-challenged settings, towards the reduction of maternal morbidity and mortality rates.

There is a clear need for research into PPC in African countries, particularly to explore novel and innovative ways to deliver PPC within existing traditional maternal and health programmes. We call on all cardiac professionals to integrate PCC into their standard of practice in order to improve pregnancy outcomes for their patients.
